# A Universally Acceptable View on the Adoption of Improved Plant Breeding Techniques

**DOI:** 10.3389/fpls.2016.01999

**Published:** 2017-01-05

**Authors:** Dennis Eriksson, Klaus H. Ammann

**Affiliations:** ^1^Department of Plant Breeding, Swedish University of Agricultural SciencesAlnarp, Sweden; ^2^Institute of Plant Sciences, University of BernBern, Switzerland

**Keywords:** genome editing, plant breeding techniques, participatory discourse, regulatory framework, regulatory triggers, biosafety assessment

There is currently a lack of regulatory clarity in the European Union (EU), as well as in other countries, for the application of genome editing in plant breeding. We argue that the development and application of genome editing may eventually bridge the regulatory discrepancy between the relatively unregulated so called conventional breeding techniques and the overregulated transgenic techniques. We also propose a participatory discourse that may help shifting focus from technique to trait in the regulation of plant breeding techniques.

## Technical progress in plant breeding

Plant breeding is the continuous endeavor to improve useful traits of crop plants by using genetic variation. Until the end of the Nineteenth century, shifts in the genetic makeup of crops mainly occurred through time-consuming phenotypic selection in the field without further knowledge of the underlying mechanisms of inheritance or the genotype-to-phenotype connection. Since the birth of the discipline of genetics and the advent of modern plant breeding though, breeders have used various scientific methods to (1) increase the available genetic variation, and (2) gain a higher level of control between deliberate genetic alterations and the resulting phenotypic traits. Mutations induced by radiation or chemicals enabled a revolution in the first mentioned, and has provided the world with at least 3240 improved varieties of all our major crops (FAO/IAEA Mutant Variety Database, 2016), whereas more recent techniques for genetic modification (GM) and genome editing have greatly enhanced the capacity both to generate genetic variation and exercise control in the breeding process.

## Recent obstacles

Since the 1980s though, progress in plant breeding techniques has faced increasing scepticism and opposition, particularly in the EU, and a somewhat misguided focus on the technique rather than the resulting trait has developed. Political maneuvering and public opinion, often loudly voiced, and exacerbated by environmental non-governmental organizations (NGO), have effectively blocked the adoption of transgenic technology in plant breeding in the EU. Only two GM crops have ever been granted authorisation for field release in the EU (1998, Monsanto, MON810 insect-resistant maize; 2010, BASF, Amflora industrial starch potato), with one (Amflora) withdrawn after only two seasons in the field. Despite numerous favorable scientific statements from the European Food Safety Authority (EFSA), other considerations than strict scientific evidence have often been given higher priority when it comes to EU decisions on field release of GM crops. At the same time, there is since long an outstanding consensus among the scientific community that GM techniques are not inherently more dangerous than any other plant breeding techniques (Kelman, 1987; National Academies of Sciences, Engineering, and Medicine, 2016). Whether or not the adoption of genome editing in plant breeding will be similarly obstructed is still an open question, as several environmental NGOs are expressing their concerns and claim that most, if not all, genome edited crops and products fall under the definition of GM organisms (GMO) and should be regulated in the same way (Greenpeace, 2015, 2016; Steinbrecher, 2015; Achterberg et al., 2016).

## Genome editing as a bridge

Recently developed genome editing techniques may nevertheless offer the possibility to bridge the dichotomy between strongly regulated transgenic techniques and the so-called conventional plant breeding techniques. Despite often using similar molecular tools as in the transgenic methodology, the products resulting from genome editing are often difficult to distinguish from those of the conventional mutation breeding methods. Our knowledge about “natural” (in this context: without human intervention) gene flow has also increased a lot lately. We now know that transgenesis between distantly related organisms occurs quite frequently in nature (Kyndt et al., 2015), and the emerging view is that of an overlapping *continuum of genetic alterations* between natural genetic variation and the wide range of tools to create genetic variation available for plant breeders (Arber, 2010). Rather than seeing plant breeding as something essentially, or increasingly, artificial, we now suggest that a more adequate view is that of mimicking natural processes, and we believe that the high level of control with genome editing techniques may facilitate this transition in perception. From the earlier relative trial-and-error approach of controlled crosses and induced mutation breeding, that cause massive alterations in the targeted genome, tools are now available for delicate modifications with surgical precision. There is a steady decrease in the amount of off-target molecular alterations (Catchpole et al., 2005; Batista et al., 2008), most recently demonstrated by minimally invasive genome editing through nanoparticle delivery of protein (Martin-Ortigosa et al., 2014) or polymer nucleic acids (PNA) (Bahal et al., 2016) that have extremely low levels of off-target effects. For all the above mentioned reasons, genome editing may be commonly appreciated as a “bridge,” both between conventional and transgenic breeding and between breeding and natural genetic and genomic processes.

## What, then, about future policy?

We need to establish a regulatory system that is both informed by science and guided by the concerns and values of all stakeholders including the public. It is indeed hard to ignore the fact that there is a substantial opposition to the use of gene technology in plant breeding, particularly in the EU. It was also rather predictable that the decade-old GMO opposition would take a similar stance on genome editing, i.e., a whole-hearted rejection from e.g., Greenpeace (see above) and Friends of the Earth (2016). However, after a decade-long deadlock on the application of GM in plant breeding in the EU, and also observing some of the reactions to genome editing, it is clear that we have to go back to the very basic questions. What the opposition, including averse policy makers, has so far failed to address is what kind of technical development would indeed be acceptable from their point of view. Also, if the public has issues with GM food products, then what kind of improved breeding techniques would actually avoid stirring peoples' fears and instead instil confidence in the professionality of researchers and breeders? To face these issues, we call for a science-guided and participatory approach to the future development and adoption of improved plant breeding techniques.

## Participatory discourse on improved plant breeding techniques

We propose that the basic approach to overcome the regulatory, public, and precautionary obstacles to technical progress in plant breeding should be to establish a technological baseline and then discuss what kind of technical progress would meet a minimum level of acceptance by everyone. The primary question would e.g., be: “*Should plant breeders be allowed in general to improve their methods and carry out their work more efficiently and with better results?*” The following question is: “*If yes; then how, i.e., under which level of biosafety assessment and other regulatory scrutiny?*” It is of course immediately clear that answering the first question with: “*No novel methods are allowed, under any circumstances*” is not a viable option. Neither is “*Under such scrutiny that all application is effectively prohibited*,” as an answer to the second question. The questions seem obvious, even naïve, yet we do not see that they have been properly addressed. It is impossible, not to mention cynical, to argue that plant breeders should not be allowed to improve the techniques they are using to generate new and better crop varieties. Let us instead use the current technological capacity as a baseline, followed by an open-minded, discursive analysis of how future plant breeding techniques may be developed and adopted. Given that genetic variation is the very corner stone of each and every plant breeding technique; what kinds of modifications to create genetic variation would be universally acceptable? This discursive analysis could cover (1) technological baseline, (2) technical progress, (3) regulatory triggers, (4) biosafety assessments, (5) the precautionary approach, (6) relevant definitions, (7) the role of science, and any other issue of relevance. Despite using a focus on techniques as a starting point, we hope that a future-looking, and multi-stakeholder discursive analysis on the (actual and potential) technical progress of breeding would eventually facilitate the disintegration of flawed regulatory concepts such as GMO, which is obsolete both from a safety point of view (Tagliabue, 2016) and from what we know about natural processes (Arber, 2010).

## The way forward

Policy making is inevitably and by necessity a multi-stakeholder process. The problem is that we have seen much argumentation over the past 25 years that does not acknowledge actual and potential benefits of gene technology, and there are repeated claims for outright bans of specific techniques in plant breeding without regard to the potential for delivering improved crop traits. The key issue now is to encourage all stakeholders, including researchers, breeders, farmers, policy makers, the public and also the GMO opposition, to discuss what kind of future technical progress in plant breeding is acceptable, and under which conditions. A future-looking discussion may offer the promise of a more neutral approach without prejudiced arguments. Regulatory models recently presented by Ricroch et al. (2016), Conko et al. (2016), and Huang et al. (2016) are very interesting since they put much stronger emphasis on the trait as trigger for biosafety assessments. We believe that these excellent models may serve as appropriate guidelines for the development of plant biotechnology legislation in the future. However, since the current EU regulatory framework for GMO to an unproportioned degree focuses on the techniques, and that applications of genome editing are threatened to also be encompassed by this framework, we regrettably acknowledge that *the techniques in themselves* will continue, at least in the near future, to constitute an important component within multi-stakeholder discussions about a future legislation for plant breeding techniques. We also agree fully with Kuzma (2016) and Ricroch et al. (2016) that a governance system with democratic legitimacy needs to be both adequately informed by science and guided by the concerns and values of all citizens. We therefore propose this complementary approach to facilitate a *quantitative-based* (e.g., level and nature of the *inevitable regulatory scrutiny*), rather than *qualitative-based* (e.g., *prohibitory* vs. *permissive*), discourse among all stakeholders for the development of a regulatory framework that is fit for any current and future plant breeding technique including genome editing. This view may result in a flexible regulation according to the rate of innovation and advances in the field of molecular breeding and also remain proportionate to the level of risk according to Podevin et al. (2012) and Wolt et al. (2016). We also cautiously predict that the transferring of genetic material between distantly related species, even across kingdoms, may be the one issue that will still prove difficult to resolve universally in a satisfactory way, as people tend to rely more on intuition than on rational and scientific reasoning when it comes to the integrity of the somewhat flawed concept of species insofar as genomic information moves beyond the idea of genes as discrete entities (Mishler and Donoghue, 1982; Mattick et al., 2010; De Queiroz, 2011). Our hope is nevertheless that the proposal will lead, little by little, to a more sensible and rational approach to plant breeding techniques in the EU, where the main focus shifts from technique to trait. In a closing remark we make a plea for flexibility in a constant risk re-evaluation according to molecular insight and also new research developments. Human, animal, and plant research often serve as inspiration to one another. The very recent example with PNA that are capable of inducing DNA repair in an animal system, and thereby catalyzing genome editing with minimally invasive treatment and extremely low rate of off-target effects (Bahal et al., 2016), demonstrates that the future regulatory framework in the EU for plant breeding techniques needs to be flexible in order to adequately accommodate novel inventions.

## Author contributions

DE prepared the initial draft. Both authors made substantial, direct and intellectual contribution to edit the work, and approved it for publication.

### Conflict of interest statement

The authors declare that the research was conducted in the absence of any commercial or financial relationships that could be construed as a potential conflict of interest.
